# Key Characteristics of Carcinogens as a Basis for Organizing Data on Mechanisms of Carcinogenesis

**DOI:** 10.1289/ehp.1509912

**Published:** 2015-11-24

**Authors:** Martyn T. Smith, Kathryn Z. Guyton, Catherine F. Gibbons, Jason M. Fritz, Christopher J. Portier, Ivan Rusyn, David M. DeMarini, Jane C. Caldwell, Robert J. Kavlock, Paul F. Lambert, Stephen S. Hecht, John R. Bucher, Bernard W. Stewart, Robert A. Baan, Vincent J. Cogliano, Kurt Straif

**Affiliations:** 1 Division of Environmental Health Sciences, School of Public Health, University of California, Berkeley, Berkeley, California, USA; 2 International Agency for Research on Cancer, Lyon, France; 3 Office of Research and Development, U.S. Environmental Protection Agency, Washington, DC, USA, and Research Triangle Park, North Carolina, USA; 4 Environmental Defense Fund, Washington, DC; 5 Department of Veterinary Integrative Biosciences, College of Veterinary Medicine and Biomedical Sciences, Texas A&M University, College Station, Texas, USA; 6 McArdle Laboratory for Cancer Research, University of Wisconsin School of Medicine and Public Health, Madison, Wisconsin, USA; 7 Masonic Cancer Center, University of Minnesota, Minneapolis, Minnesota, USA; 8 National Toxicology Program, National Institute of Environmental Health Sciences, National Institutes of Health, Department of Health and Human Services, Research Triangle Park, North Carolina, USA; 9 Faculty of Medicine, University of New South Wales, Sydney, New South Wales, Australia

## Abstract

**Background:**

A recent review by the International Agency for Research on Cancer (IARC) updated the assessments of the > 100 agents classified as Group 1, carcinogenic to humans (IARC Monographs Volume 100, parts A–F). This exercise was complicated by the absence of a broadly accepted, systematic method for evaluating mechanistic data to support conclusions regarding human hazard from exposure to carcinogens.

**Objectives:**

and Methods: IARC therefore convened two workshops in which an international Working Group of experts identified 10 key characteristics, one or more of which are commonly exhibited by established human carcinogens.

**Discussion:**

These characteristics provide the basis for an objective approach to identifying and organizing results from pertinent mechanistic studies. The 10 characteristics are the abilities of an agent to 1) act as an electrophile either directly or after metabolic activation; 2) be genotoxic; 3) alter DNA repair or cause genomic instability; 4) induce epigenetic alterations; 5) induce oxidative stress; 6) induce chronic inflammation; 7) be immunosuppressive; 8) modulate receptor-mediated effects; 9) cause immortalization; and 10) alter cell proliferation, cell death, or nutrient supply.

**Conclusion:**

We describe the use of the 10 key characteristics to conduct a systematic literature search focused on relevant end points and construct a graphical representation of the identified mechanistic information. Next, we use benzene and polychlorinated biphenyls as examples to illustrate how this approach may work in practice. The approach described is similar in many respects to those currently being implemented by the U.S. EPA’s Integrated Risk Information System Program and the U.S. National Toxicology Program.

**Citation:**

Smith MT, Guyton KZ, Gibbons CF, Fritz JM, Portier CJ, Rusyn I, DeMarini DM, Caldwell JC, Kavlock RJ, Lambert P, Hecht SS, Bucher JR, Stewart BW, Baan R, Cogliano VJ, Straif K. 2016. Key characteristics of carcinogens as a basis for organizing data on mechanisms of carcinogenesis. Environ Health Perspect 124:713–721; http://dx.doi.org/10.1289/ehp.1509912

## Introduction

Recently, the International Agency for Research on Cancer (IARC) completed a review of all its Group 1 human carcinogens and updated information on tumor sites and mechanisms of carcinogenesis (IARC Monograph Volume 100A–F) (http://monographs.iarc.fr/ENG/Monographs/PDFs/index.php). About half of the agents classified in Group 1 had been last reviewed > 25 years ago, before mechanistic studies became prominent in evaluations of carcinogenicity. In addition, more recent studies have demonstrated that many cancer hazards reported in earlier studies were later observed to also cause cancer in other organs or through different exposure scenarios (Cogliano et al. 2011).

In compiling and updating the information for Volume 100A–F, two overarching issues became apparent. First, no broadly accepted systematic method for identifying, organizing, and summarizing mechanistic data for the purpose of decision making in cancer hazard identification was readily available. Second, the agents documented and listed as human carcinogens showed a number of characteristics that are shared among many carcinogenic agents. Many human carcinogens act via multiple mechanisms causing various biological changes in the multistage process of carcinogenesis. Indeed, cancer was once described by reference to causative agents, with multistage development of tumors being characterized through the impact of particular chemicals described as initiators and promoters of cancer. Subsequently, multistage development of cancer was identified with morphological change being correlated with genetic alterations. The more recent description by Hanahan and Weinberg of hallmarks of cancer is predicated not on morphology or the impact of carcinogens, but on changes in gene expression and cell signaling (Hanahan and Weinberg 2011). These hallmarks are the properties of cancer cells and neoplasms, and are not characteristic of the agents that cause cancer. Tumors attributable to chemical carcinogens may be distinct by mutational analysis (Westcott et al. 2015), but all neoplasms exhibit the hallmarks. A recent computational toxicology study has shown that chemicals that alter the targets or pathways among the hallmarks of cancer are likely to be carcinogenic (Kleinstreuer et al. 2013). In addition, a series of reviews in *Carcinogenesis* by members of the Halifax Project Task Force used the hallmarks framework to identify the carcinogenic potential of low doses and mixtures of chemicals (Harris 2015).

In 2012, participants at two workshops convened by the IARC in Lyon, France, extensively debated the mechanisms by which agents identified as human carcinogens (Group 1) produce cancer. The participants concluded that these carcinogens frequently exhibit ≥ 1 of 10 key characteristics (Table 1). Herein we describe these 10 key characteristics and discuss their importance in carcinogenesis. These characteristics are properties that human carcinogens commonly show and can encompass many different types of mechanistic end points. They are not mechanisms in and of themselves nor are they adverse outcome pathways.

**Table 1:** Key characteristics of carcinogens.

Characteristic	Examples of relevant evidence
1. Is electrophilic or can be metabolically activated	Parent compound or metabolite with an electrophilic structure (e.g., epoxide, quinone), formation of DNA and protein adducts
2. Is genotoxic	DNA damage (DNA strand breaks, DNA–protein cross-links, unscheduled DNA synthesis), intercalation, gene mutations, cytogenetic changes (e.g., chromosome aberrations, micronuclei)
3. Alters DNA repair or causes genomic instability	Alterations of DNA replication or repair (e.g., topoisomerase II, base-excision or double-strand break repair)
4. Induces epigenetic alterations	DNA methylation, histone modification, microRNA expression
5. Induces oxidative stress	Oxygen radicals, oxidative stress, oxidative damage to macromolecules (e.g., DNA, lipids)
6. Induces chronic inflammation	Elevated white blood cells, myeloperoxidase activity, altered cytokine and/or chemokine production
7. Is immunosuppressive	Decreased immunosurveillance, immune system dysfunction
8. Modulates receptor-mediated effects	Receptor in/activation (e.g., ER, PPAR, AhR) or modulation of endogenous ligands (including hormones)
9. Causes immortalization	Inhibition of senescence, cell transformation
10. Alters cell proliferation, cell death or nutrient supply	Increased proliferation, decreased apoptosis, changes in growth factors, energetics and signaling pathways related to cellular replication or cell cycle control, angiogenesis
Abbreviations: AhR, aryl hydrocarbon receptor; ER, estrogen receptor; PPAR, peroxisome proliferator–activated receptor. Any of the 10 characteristics in this table could interact with any other (e.g., oxidative stress, DNA damage, and chronic inflammation), which when combined provides stronger evidence for a cancer mechanism than would oxidative stress alone.	

Further, we describe how the 10 key characteristics can provide a basis for systematically identifying, organizing, and summarizing mechanistic information as part of the carcinogen evaluation process. The U.S. Environmental Protection Agency (EPA) and the National Toxicology Program (NTP) in the United States, as well as the IARC internationally, have recognized a need for such an approach (Rooney et al. 2014). The U.S. National Research Council (NRC) emphasized the need for consistent, transparent, systematic approaches for the identification, evaluation, and integration of data in the U.S. EPA’s Integrated Risk Information System (IRIS) assessments of carcinogens and elsewhere in human health hazard assessments (NRC 2014).

Progress in the systematic evaluation of published evidence on the adverse health effects of environmental agents has been made through application of methods developed by evidence-based medicine (Koustas et al. 2014). However, mechanistic study databases present a challenge to systematic reviews in that the studies are typically both numerous and diverse, reporting on a multitude of end points and toxicity pathways. One recent example of a systematic approach searched for studies on end points relevant to nine cancer-related mechanistic categories in identifying and presenting mechanistic evidence on di(2-ethylhexyl) phthalate, a chemical with a complex database of > 3,000 research papers (Kushman et al. 2013). In this publication, the categories of mechanistic evidence were identified from a compendium of published reviews. This approach may be difficult to translate to agents with controversial or limited mechanistic evidence. It also would not permit comparisons across agents, including attempts to understand similarities or differences with human carcinogens. Further, it may be biased against the most recent mechanistic and molecular epidemiology studies that have not been the subject of a prior expert review.

To facilitate a systematic and uniform approach to organizing mechanistic data relevant to carcinogens, we propose use of the 10 key characteristics of human carcinogens as a basis for identifying and categorizing scientific findings relevant to cancer mechanisms when assessing whether an agent is a potential human carcinogen. A significant advantage of this approach is that it would encompass a wide range of end points of known relevance to carcinogenesis as identified through examination of the IARC Monographs on Group 1 carcinogens. Mechanistic topics can be included regardless of whether they have been the subject of prior expert reviews of any particular chemical. This should introduce objectivity that could reduce reliance on expert opinion, as well as facilitate comparisons across agents. Moreover, at its essence, the approach may afford a broad consideration of the mechanistic evidence rather than focusing narrowly on independent mechanistic hypotheses or pathways in isolation.

Herein, we demonstrate the applicability of this proposed systematic strategy for searching and organizing the literature using benzene and polychlorinated biphenyls (PCBs) as examples. The mechanistic study database for both of these chemicals is large, comprising > 1,800 studies for benzene and almost 3,900 for PCBs, many with multiple mechanistic end points. We conducted systematic literature searches for end points pertinent to the 10 key characteristics of human carcinogens, using literature trees to indicate the human and experimental animal studies that reported end points relevant to each characteristic. To further indicate their potential contribution to benzene and PCB carcinogenesis, we organized the characteristics into a graphical network representative of an overall mechanistic pathway.

Several recent IARC Monographs (e.g., Guyton et al. 2015; Loomis et al. 2015) have applied the 10 key characteristics described here for a variety of agents and organized the literature search results into flow diagrams. Overall, this categorization facilitated objective consideration of the relevant mechanistic information, thereby advancing analyses of hypothesized mechanisms and toxicity pathways. Because mechanistic data may provide evidence of carcinogenicity, and can play a role in up- or downgrading an evaluation based on cancer findings in animals, we suggest that this systematic approach to organizing the available data will assist future IARC Working Groups and other agencies in evaluating agents as potential human carcinogens, especially in the absence of convincing epidemiological data on cancer in humans.

### Description of the Key Characteristics of Carcinogens

The number of ways by which agents contribute to carcinogenesis can be extensive if all biochemical or molecular end points are considered. However, these mechanisms can be grouped into a limited number of categories (e.g., genotoxicity, immunosuppression). Guyton et al. (2009) described 15 types of “key events” associated with human carcinogens that collectively represented many carcinogenic mechanisms. The experts present at the first of the IARC meetings in 2012 originally identified 24 mechanistic end points with several subcategories in each. This number of end points was considered too impractical as a guide for categorizing the literature, and the Working Group merged these categories into 10 at the second meeting in 2012, concluding that human carcinogens commonly show ≥ 1 of the 10 key characteristic properties listed in Table 1. These represent the majority of established properties of human carcinogens as described below.

### Characteristic 1: Is Electrophilic or Can Be Metabolically Activated to Electrophiles

Electrophiles are electron-seeking molecules that commonly form addition products, commonly referred to as adducts, with cellular macromolecules including DNA, RNA, lipids, and proteins. Some chemical carcinogens are direct-acting electrophiles, whereas others require chemical conversion within the body (Salnikow and Zhitkovich 2008) or biotransformation by enzymes in a process termed metabolic activation (Miller 1970). Examples of direct-acting electrophilic carcinogens include sulfur mustards and ethylene oxide (Batal et al. 2014; Grosse et al. 2007; IARC 2008; Rusyn et al. 2005). The classic examples of chemical agents that require metabolic activation to become carcinogenic include polycyclic aromatic hydrocarbons, aromatic amines, *N*-nitrosamines, aflatoxins, and benzene, which by themselves are relatively inert (Slaga et al. 1980; Smith 1996). A number of enzymes, including cytochrome P450s, flavin mono-oxygenase, prostaglandin synthase, and various peroxidases, can biotransform relatively inert chemical compounds to potent toxic and carcinogenic metabolites or reactive intermediates (Hecht 2012; O’Brien 2000). The ability to form adducts on nucleic acids and proteins is a common property of these inherently electrophilic and/or metabolically activated human carcinogens (Ehrenberg 1984).

### Characteristic 2: Is Genotoxic

The term “genotoxic” (Ehrenberg et al. 1973) refers to an agent that induces DNA damage, mutation, or both. DNA damage can be spontaneous in origin through errors of nucleic acid metabolism or can be induced by endogenous or exogenous agents. In some cases the exogenous agents may also be generated endogenously, such as formaldehyde and acetaldehyde, producing a background level of DNA damage. Examples of DNA damage include DNA adducts (a molecule bound covalently to DNA), DNA strand breaks (breaks in the phosphodiester bonds), DNA crosslinks, and DNA alkylation. DNA damage by itself is not a mutation and generally does not alter the linear sequence of nucleotides (or bases) in the DNA, whereas a mutation is a change in the DNA sequence and usually arises as the cell attempts to repair the DNA damage (Shaughnessy and DeMarini 2009).

Mutations can be classified into three groups based on their location or involvement in the genome. Gene or point mutations are changes in nucleotide sequence within a gene (e.g., base substitutions, frameshifts, and small deletions/duplications). Chromosomal mutations are changes in nucleotide sequence that extend over multiple genes (e.g., chromosome aberrations, translocations, large deletions, duplications, insertions, inversions, or micronuclei due to chromosome breakage). Genomic mutations involve the duplication or deletion of nucleotide sequences of an entire chromosome, an example of which is aneuploidy or formation of micronuclei that contain a centromere. A large proportion of Group 1 carcinogens are genotoxic, as documented in IARC Monographs Volume 100 A–F.

### Characteristic 3: Alters DNA Repair or Causes Genomic Instability

Normal cells avoid deleterious mutations by replicating their genomes with high accuracy. However, the fidelity of DNA replication can vary widely depending on the DNA polymerase involved, introducing the possibility of error. Indeed, most spontaneous mutations are caused by polymerase error (Preston et al. 2010). The nature of the error, the flanking sequence, the presence of DNA damage, and the ability to correct errors all affect the outcome of this process (Arana and Kunkel 2010). As a consequence, defects in processes that determine DNA-replication fidelity can confer strong mutator phenotypes that result in genomic instability. Thus, carcinogens may act not only by producing DNA damage directly, but also by altering the processes that control normal DNA replication or repair of DNA damage. Examples include the inhibition of DNA repair by cadmium (Candéias et al. 2010) and formaldehyde (Luch et al. 2014).

Genomic instability is a well-recognized feature of many cancers (Bielas et al. 2006) and is considered to be one of the enabling characteristics of cancer (Hanahan and Weinberg 2011). Cells exposed to ionizing radiation have genetic instability that is a relatively late-occurring event that appears several cell generations after irradiation and results in a reduced ability to replicate the genotype faithfully (Kadhim et al. 2013). The events indicating genomic instability include chromosome aberrations, gene mutations, microsatellite instability, and apoptosis. These events are observed after exposure to arsenic (Bhattacharjee et al. 2013) and cadmium (Filipic 2012).

### Characteristic 4: Induces Epigenetic Alterations

The term “epigenetic” refers to stable changes in gene expression and chromatin organization that are not caused by changes in the DNA sequence itself and can be inherited over cell divisions (Herceg et al. 2013). Epigenetic phenomena, including changes to the DNA methylome and chromatin compaction states, along with histone modification can impact the carcinogenic process by affecting gene expression and DNA repair dynamics (Herceg et al. 2013). A wide range of carcinogens have been shown to deregulate the epigenome, and it has been suggested that their mechanism may involve disruption of epigenetic mechanisms (Pogribny and Rusyn 2013). However, evidence for a causal role of epigenetic changes in cancer caused by Group 1 agents was considered to be limited in Volume 100, and the impact of many agents on the epigenome was considered to be a secondary mechanism of carcinogenesis (Herceg et al. 2013). Herceg et al. (2013) have described a wealth of studies demonstrating the impact of carcinogens on epigenetic mechanisms. Most carcinogens (even those reviewed for Volume 100) were evaluated by IARC Working Groups before new data on their epigenetic effects became available (Chappell et al. 2016). This evolving area will generate new mechanistic data in the years to come.

### Characteristic 5: Induces Oxidative Stress

Many carcinogens are capable of influencing redox balance within target cells. If an imbalance occurs, favoring formation of reactive oxygen and/or nitrogen species at the expense of their detoxification, this is referred to as oxidative stress. Reactive oxygen species and other free radicals arising from tissue inflammation, xenobiotic metabolism, interruption of mitochondrial oxidative phosphorylation (Figueira et al. 2013), or reduced turnover of oxidized cellular components may play key roles in many of the processes necessary for the conversion of normal cells to cancer cells. However, oxidative stress is not unique to cancer induction and is associated with a number of chronic diseases and pathological conditions—for example, cardiovascular disease (Kayama et al. 2015), neurodegenerative disease (Chen et al. 2016), and chronic inflammation (Suman et al. 2015). Oxidative stress is also a common occurrence in neoplastic tissue and can be part of the tumor environment (Suman et al. 2015).

Oxidative damage is considered a major factor in the generation of mutations in DNA, and > 100 different types of oxidative DNA damage have been identified (Klaunig et al. 2011). At least 24 base modifications are produced by reactive oxygen species, as well as DNA–protein crosslinks and other lesions (Berquist and Wilson 2012), all potentially leading to genomic instability. Oxidative damage to DNA can lead to point mutations, deletions, insertions, or chromosomal translocations, which may cause oncogene activation and tumor suppressor gene inactivation, and potentially initiate or promote carcinogenesis (Berquist and Wilson 2012; Klaunig et al. 2011). Thus, the induction of oxygen radical–induced cellular injury is a characteristic of a set of diverse carcinogens, including radiation, asbestos, and carcinogenic infectious agents.

### Characteristic 6: Induces Chronic Inflammation

Chronic inflammation from persistent infections, such as that caused by *Helicobacter pylori*, as well as that produced by chemical agents including silica or asbestos fibers, has been associated with several forms of cancer (Grivennikov et al. 2010). Indeed, inflammation has been hypothesized to contribute to multiple aspects of cancer development and progression (Trinchieri 2012) and is an enabling hallmark of cancer (Hanahan and Weinberg 2011). Inflammation acts by both intrinsic and extrinsic pathways. Persistent infection and chronic inflammation disrupt local tissue homeostasis and alter cell signaling, leading to the recruitment and activation of inflammatory cells. These constitute extrinsic pathways linking inflammation to cancer (Multhoff and Radons 2012). On the other hand, intrinsic pathways driven by activation of proto-oncogenes in pre-neoplastic and neoplastic cells recruit host-derived inflammatory cells that accelerate tumor promotion and progression (Grivennikov et al. 2010). Because strong links exist between inflammation and the induction of oxidative stress and genomic instability, it may be difficult to separate out the importance of each of these mechanisms.

### Characteristic 7: Is Immunosuppressive

Immunosuppression is a reduction in the capacity of the immune system to respond effectively to foreign antigens, including antigens on tumor cells. Persistent immunosuppression presents a risk of cancer, especially excess risk for lymphoma. For example, immunosuppression poses a significant risk when it is accompanied by continuing exposure to foreign antigens, such as in people with organ transplants, or when it occurs in individuals who are latently infected with a carcinogenic virus (Hartge and Smith 2007; Smith et al. 2004). Immune suppression differs from other mechanisms of carcinogenesis in that agents that cause immunosuppression may not directly transform normal cells into potential tumor cells. Potentially neoplastic cells that arise naturally, or that have been transformed by other carcinogens acting by a mechanism such as genotoxicity or by the various mechanisms of action associated with carcinogenic viruses, escape immune surveillance in immunosuppressed individuals. As a result, survival of these cells and their replication to form tumors is greatly facilitated by immune suppression. Several carcinogens act entirely or largely by immunosuppression, often in concert with other Group 1 agents, especially oncogenic infectious agents. The Group 1 agents that act by immunosuppression include human immunodeficiency virus (HIV-1) and the immunosuppressive drug cyclosporin (Rafferty et al. 2012).

### Characteristic 8: Modulates Receptor-Mediated Effects

Numerous carcinogens act as ligands to receptor proteins, including menopausal hormone therapy, 2,3,7,8-tetrachlorodibenzo-*p*-dioxin and PCBs (Wallace and Redinbo 2013). Receptor-mediated activation broadly falls into two categories: *a*) intracellular activation, mediated by nuclear receptors that translocate into the nucleus and act on DNA as transcription factors (Aranda and Pascual 2001); and *b*) activation of cell surface receptors that induce signal-transduction pathways resulting in biological responses that involve a variety of protein kinases (Griner and Kazanietz 2007). Most exogenous agents act as agonists by competing for binding with an endogenous ligand; however, there are also receptors for which few or no endogenous ligands have been identified, such as the aryl hydrocarbon (Ah) receptor (Baek and Kim 2014; Ma 2011). Receptor-mediated activation most often results in changes in gene transcription. Molecular pathways that are regulated through ligand-receptor interaction and are most relevant to carcinogenesis include cell proliferation (e.g., stimulation of the normal proliferative pathways, as is the case for estrogen-dependent tissues and hormone therapy), xenobiotic metabolism, apoptosis, as well as modulation of the bioavailability of endogenous ligands by affecting biosynthesis, bioactivation, and degradation (Rushmore and Kong 2002).

### Characteristic 9: Causes Immortalization

Several human DNA and RNA viruses, including various human papillomaviruses, Epstein-Barr virus, Kaposi sarcoma–associated herpes virus, hepatitis B virus, hepatitis C virus, HIV, Merkel cell polyomavirus (MCPyV), and human T-lymphotropic virus type 1 (HTLV-1) are carcinogenic to humans (Bouvard et al. 2009). These viruses have evolved multiple molecular mechanisms to disrupt specific cellular pathways to facilitate aberrant replication. Although oncogenic viruses belong to different families, their strategies in human cancer development show many similarities and involve viral-encoded oncoproteins targeting the key cellular proteins that regulate cell growth (Saha et al. 2010). Recent studies show that virus and host interactions also occur at the epigenetic level (Allday 2013). The result of these viral effects is to immortalize the target tissue cells such that they are not subject to the Hayflick limit, the point at which cells can no longer divide due to DNA damage or shortened telomeres (Klingelhutz 1999). For example, the human papilloma virus type 16 (HPV-16) *E6* and *E7* oncogenes are selectively retained and expressed in cervical carcinomas, and expression of *E6* and *E7* is sufficient to immortalize human cervical epithelial cells (Yugawa and Kiyono 2009).

### Characteristic 10: Alters Cell Proliferation, Cell Death, or Nutrient Supply

There are at least three scenarios related to carcinogenesis in which alterations in cellular replication and/or cell-cycle control have been described. One invokes the predisposition for unrepaired DNA damage leading to cancer-causing mutations in replicating cells; another has attempted to identify sustained replication as a key mechanistic event; and a third describes the ability of a transformed cell to escape normal cell-cycle control and to continue replication. A component common to all three scenarios is the evasion of apoptosis or other terminal programming, including autophagy, in at least a proportion of the cell population (Ryter et al. 2014).

Necrotic cell death releases pro-inflammatory signals into the surrounding tissue microenvironment, recruiting inflammatory immune cells to the site of trauma, which can enhance cancer-cell proliferation and promote cancer metastasis (Coussens and Pollard 2011; Coussens et al. 2013; Pollard 2008). In contrast, various forms of apoptosis and autophagy (Galluzzi et al. 2015) have the opposite effect by removing potentially cancerous cells from a population before they acquire the changes permitting malignancy. Many agents affect necrosis, apoptosis, and/or autophagy and can have profoundly divergent effects on cancer induction in different tissues.

In addition to cell death caused directly by agent toxicity, cells may die within a tumor as a result of an impaired nutrient supply. Neoplastic cell numbers can increase exponentially, quickly outstripping the supply capabilities of the existing tissue vasculature. Neoangiogenesis, in which new blood vessels grow into a tumor, is key to providing this supply of nutrients. Thus, agents that promote or inhibit angiogenesis will promote or delay tumor growth (Hu et al. 2015).

Cancer cells also usually show quite different cellular energetics, relying on glycolysis for energy even under aerobic conditions (Rajendran et al. 2004). Although a likely consequence of mutation and altered gene expression rather than a cancer-inducing mechanism, any modification of cellular energetics may reflect an important cancer-relevant switch in the cell’s or tissue’s metabolic state.

## Using the Key Characteristics to Systematically Identify, Organize, and Summarize Mechanistic Information

### Step 1: Identifying the Relevant Information

The starting point for systematic evaluation is to conduct comprehensive searches of the peer-reviewed literature aimed at identifying mechanistic data (Kushman et al. 2013). The searches can be constructed to address a series of study questions in the PECO (population, exposure, comparator, and outcomes) framework (Higgins and Green 2011) wherein end points associated with the key characteristics are identified. Specifically, the question to be answered by the searches is “Does exposure to the agent induce end points associated with one or more specific key characteristic properties of carcinogens?” The population (humans and any relevant experimental systems), exposure (the agent and relevant metabolites), and comparator (the unexposed comparison group or condition) should be sufficiently broad to identify a range of available mechanistic data informative of the overall evaluation of carcinogenic hazard. This approach thus entails comprehensive, targeted literature searches using appropriate medical search heading (MeSH) terms and key words to identify evidence on the 10 key characteristics for the agent(s) or exposure(s) under evaluation.

Additional complementary literature searches may incorporate terms for the agent and its metabolites, alone or in combination with broad terms for carcinogenicity or related effects. For instance, because U.S. EPA IRIS toxicological reviews also encompass a range of non-cancer toxicities, “top-down” broad literature searches aimed at comprehensively identifying studies on all potential toxic effects of an agent are employed (NRC 2014; U.S. EPA 2014). These comprehensive searches of peer-reviewed literature are supplemented by examining past IARC Monographs or other authoritative reviews, databases (e.g., PubChem), and peer-reviewed government reports can also be systematically searched. The search terms used and literature retrieved can be documented (e.g., using MyNCBI, which saves searches of the National Center for Biotechnology database, or https://hawcproject.org/).

### Step 2: Screening and Organizing the Results

Based on title and abstract review, studies identified initially are excluded if no data on the chemical or a metabolite are reported, or if no data on toxicological or other cancer-related effects of the chemical are provided. For example, a study on levels of a chemical, but not effects of the chemical, would be excluded. Included studies are then organized by the population (human or experimental systems) and by the end points associated with the 10 key characteristics (Table 1). Studies relevant to toxicokinetics (covering absorption, distribution, metabolism, and excretion) are also identified. Additionally, authoritative, comprehensive review articles are identified, as are studies reporting toxicological end points in cancer target and non-target tissues. These may include morphological evaluations pertaining to the dysfunction of organs, tissues, and cells. Importantly, studies reporting end points that are relevant to multiple characteristics may fall under several categories.

**Figure 1:**
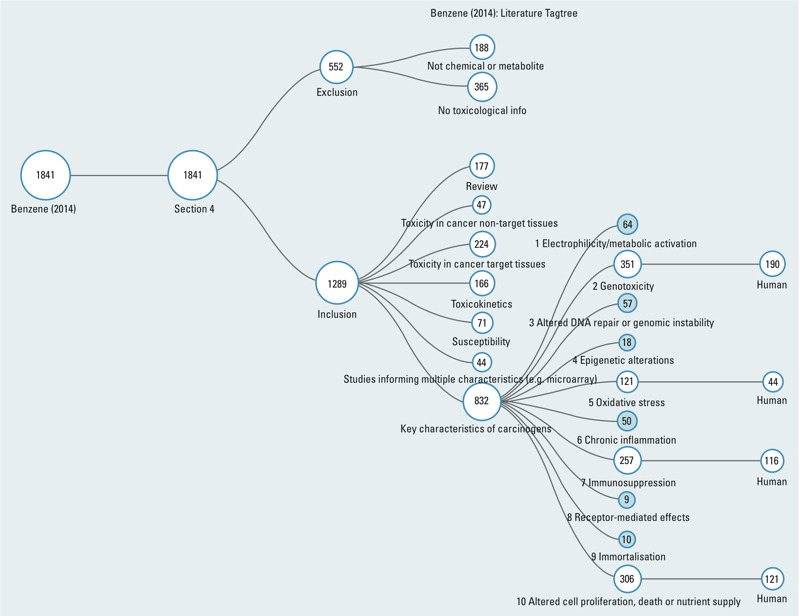
Literature flow diagram, illustrating the systematic identification and categorization process for benzene mechanistic studies. Using appropriate MeSH terms and key words, targeted literature searches were conducted for the 10 key characteristics using online tools available from the HAWC Project (https://hawcproject.org/). Section 4 refers to the location of the discussion of mechanistic data within the IARC Monograph structure (http://monographs.iarc.fr/ENG/Preamble/currentb4studiesother0706.php). All inclusion categories were expanded to document the number of studies attributed to each, down to the individual key characteristic level, which were expanded to illustrate human information when > 100 total studies were identified. Less frequently encountered key characteristic categories (blue-shaded circles) were left unexpanded for clarity. “Human” refers to both humans exposed *in vivo* and human cells exposed *in vitro*.

To illustrate these two steps, targeted literature searches were conducted to identify end points for the effects of benzene pertinent to the 10 key characteristics, in populations comprising humans or experimental systems. The literature searches were conducted using the Health Assessment Workplace Collaborative (HAWC) Literature Search tool (https://hawcproject.org/), documenting the search terms, sources, and articles retrieved. Following title and abstract review, studies were excluded if they were not about benzene or its metabolites, or if they reported no data on toxicological end points. Included studies were further sorted into categories representing the 10 key characteristics based on the mechanistic end points and species evaluated (i.e., human *in vivo*, human *in vitro*, mammalian *in vivo*, mammalian *in vitro*, nonmammalian; Figure 1). The figure also identifies reviews, gene expression studies, and articles relevant to toxicokinetics, toxicity, or susceptibility.

### Step 3: Using the Key Characteristics to Synthesize Mechanistic Information and to Develop Adverse-Outcome Networks

**Figure 2:**
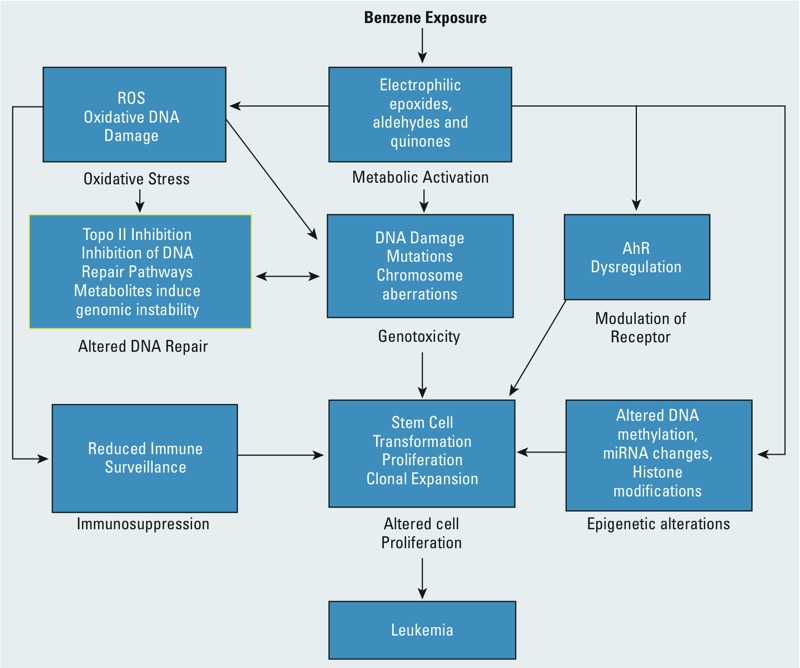
An overview of how benzene induces eight of the key characteristics in a probable mechanism of carcinogenicity. A full review of these mechanistic data is given by McHale et al. (2012), from which this figure was adapted.

It is increasingly evident that multiple biological alterations or sets of different perturbations are necessary to convert a normal cell to a transformed cell and ultimately a tumor (Hanahan and Weinberg 2011). Carcinogens appear to affect this complex process in various ways and can act through multiple mechanisms to induce cancer and other adverse health outcomes (Goodson et al. 2015; Guyton et al. 2009). Using the 10 key characteristics as a basis, the collected information can be organized to form hypotheses and evaluate the evidentiary support for mechanistic events as a function of relevant aspects (e.g., dose, species, temporality) (Guyton et al. 2009). The diverse and complex mechanistic end points elicited by benzene can then be organized into an overview inclusive of multiple alterations and any linkages thereof (Figure 2). The resulting overview can provide guidance for further assessments of the literature, including dose relevance, species relevance, and temporality of events. This additional detailed information can then be used to produce proposed mechanisms or adverse outcome pathway networks as described by McHale et al. (2012) and the EPA’s NexGen Risk Assessment Report (U.S. EPA 2014). We note that there is evidence that benzene is associated with 8 of the 10 key characteristics we have described.

**Figure 3:**
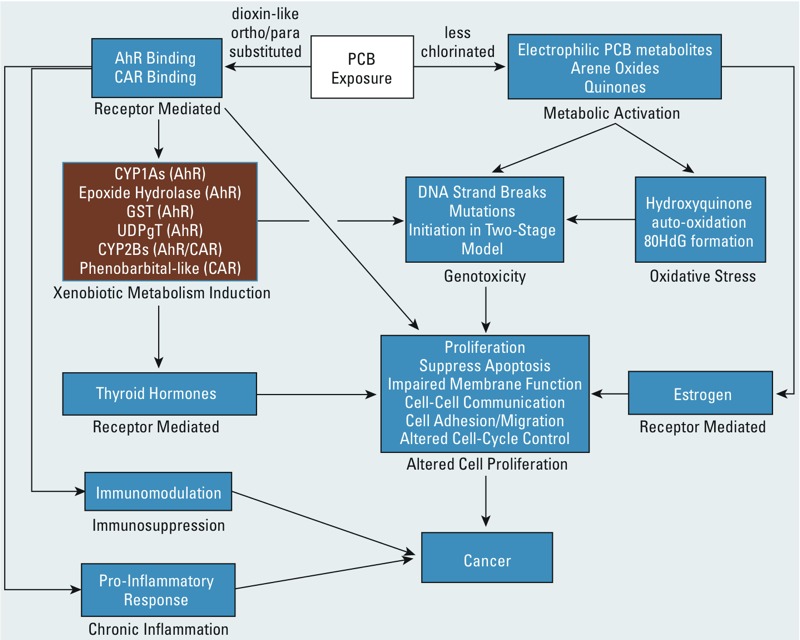
An overview of how polychlorinated biphenyls (PCBs) may induce seven key characteristics in their carcinogenicity (Lauby-Secretan et al. 2013). Highly chlorinated PCBs act as ligands for the aryl hydrocarbon receptor (AhR) and other receptors activating a large number of genes in a tissue- and cell-specific manner that can lead to cell proliferation, apoptosis, and other effects that influence cancer risk. Less chlorinated PCBs can be activated to electrophilic metabolites, such as arene oxides and quinones, which can cause genotoxic effects and induce oxidative stress. Receptor binding to CAR (constitutive androstane receptor) and AhR (a key characteristic) leads to xenobiotic metabolism induction (not a key characteristic; brown box) that in turn leads to genotoxicity and other key characteristics.

Figure 3 presents a similar overview for PCBs based on data from IARC Monograph Volume 107 (IARC 2015). In summarizing the mechanistic evidence, this Monograph Working Group indicated that PCBs may induce up to 7 of the 10 key characteristics in producing carcinogenicity (Lauby-Secretan et al. 2013). The less chlorinated PCBs are associated with key characteristics similar to benzene (metabolic activation, DNA damage, cellular proliferation), whereas the dioxin-like PCBs are associated primarily with receptor-mediated activities.

Recently, using this same approach, the Working Groups of IARC Monograph Volume 112 and Volume 113 (in progress) concluded that strong mechanistic evidence exists for five key characteristics being involved in malathion carcinogenicity (i.e., genotoxicity, oxidative stress, inflammation, receptor-mediated effects, and cell proliferation or death), three in DDT carcinogenicity (i.e., immunosuppression, receptor-mediated effects and oxidative stress), and two each for diazinon and glyphosate (i.e., genotoxicity and oxidative stress), providing evidence to support their classification as probable human carcinogens in Group 2A (Guyton et al. 2015; Loomis et al. 2015).

## Discussion and Conclusions

Identification and incorporation of important, novel scientific findings providing insights into cancer mechanisms is an increasingly essential aspect of carcinogen hazard identification and risk assessment. Systematic approaches are needed to organize the available mechanistic data relevant to the overall evaluation of the carcinogenic hazard of an agent. Information to support the identification of 10 key characteristics of human carcinogens was obtained during the Volume 100 Monographs and two subsequent expert workshops. These characteristics, although not necessarily representing mechanisms themselves, provide the rationale for an objective approach to identifying and organizing relevant mechanistic data. Using literature collected previously by others as well as by us, we have categorized the literature data according to the 10 characteristics for benzene and PCBs. This approach identified pertinent positive literature for 8 of the 10 key characteristics on benzene and 7 for PCBs, thereby providing a practical, objective method for organizing the large mechanistic literature associated with these chemicals.

This approach also lays the groundwork for a structured evaluation of the strength of the mechanistic evidence base, and therefore its utility in supporting hazard classifications. In the IARC Monographs the strength of the evidence that any carcinogenic effect observed is attributable to a particular mechanism is evaluated using the terms “weak,” “moderate,” or “strong” (http://monographs.iarc.fr/ENG/Preamble/index.php). In general, the strongest indications that a particular mechanism operates in humans derive from data obtained in exposed humans or in human cells *in vitro*. Data from experimental animals can support a mechanism by findings of consistent results and from studies that challenge the hypothesized mechanism experimentally. Other considerations include whether multiple mechanisms might contribute to tumor development, whether different mechanisms might operate in different dose ranges, whether separate mechanisms might operate in humans and experimental animals, and whether a unique mechanism might operate in a susceptible group. The possible contribution of alternative mechanisms must be considered before concluding that tumors observed in experimental animals are not relevant to humans. An uneven level of experimental support for different mechanisms may reflect that disproportionate resources have been focused on investigating a favored mechanism. All of these factors make assignment of descriptors such as “strong” to the mechanistic evidence challenging; but recent experience with two IARC Monograph meetings suggest that the weighing of the evidence on the basis of the 10 key characteristics focuses the group discussion on the available science and allows rapid consensus to be reached regardless of the strength of the evidence base (Guyton et al. 2015; Loomis et al. 2015).

Because the literature search and categorization approach described herein is comprehensive, it may aid consideration of the overall strength of the mechanistic database according to these principles. In particular, it is inclusive of diverse mechanistic evidence, enabling support for divergent or related mechanisms from human and experimental systems to be identified. Moreover, the literature support for end points relevant to specific mechanisms can be evaluated in an integrated manner when the mechanism is complex. Additionally, comparisons across agents will be facilitated, including evaluation of any similarities or differences in the pattern of key characteristics with agents that are currently classified.

As this approach is carried forward, we hope it will facilitate the objective identification of mechanistic data for consideration in the context of epidemiology, animal bioassay, or other types of evidence (e.g., studies in model organisms or *in vitro* assays) when classifying agents with regard to carcinogenic hazard. Equally important is to consider whether key characteristics of carcinogens are apparent upon exposures that are relevant to human health (Thomas et al. 2013). Overall, these developments will aid advancement of future evaluations of newly introduced agents, including those for which mechanistic data provide the primary evidence of carcinogenicity.
